# Physical Activity Levels and Predictors during COVID-19 Lockdown among Lebanese Adults: The Impacts of Sociodemographic Factors, Type of Physical Activity and Work Location

**DOI:** 10.3390/healthcare11142080

**Published:** 2023-07-21

**Authors:** Mireille Harmouche-Karaki, Maya Mahfouz, Pascale Salameh, Nour El Helou

**Affiliations:** 1 Department of Nutrition, Faculty of Pharmacy, Saint Joseph University, Beirut 1107 2180, Lebanon; mireille.harmouche@usj.edu.lb (M.H.-K.); nour.helou@usj.edu.lb (N.E.H.); 2 School of Medicine, Lebanese American University, Byblos 1102 2801, Lebanon; psalameh@ul.edu.lb; 3 Institut National de Santé Publique d'Épidémiologie Clinique et de Toxicologie-Liban (INSPECT-LB), Beirut 1103 2180, Lebanon; 4 Department of Primary Care and Population Health, University of Nicosia Medical School, Nicosia 2417, Cyprus; 5 Faculty of Pharmacy, Lebanese University, Hadat 1533, Lebanon

**Keywords:** exercise, sedentary behavior, quarantine, machine learning, workplace

## Abstract

Background: Although effective against COVID-19, national lockdowns have several deleterious behavioral and health effects, including physical inactivity. The objective of this study is to assess physical activity (PA) levels during lockdown and the predictors of PA among Lebanese adults, while comparing classical statistics to machine learning models. Methods: Data were collected using an online questionnaire, with PA being evaluated through the “International Physical Activity Questionnaire” (IPAQ)—long form. Machine learning models were applied to predict total PA ≥ 600 MET·min/week. Results: Among 795 participants, while 67.5% auto-declared a decrease in PA level during lockdown, 36.2% did not meet the minimum recommendations for PA. Multivariate analysis showed that participants who went to their workplace during lockdown had significantly higher total and job-related PA, higher walking and moderate PA, and lower sitting time. PA level and intensity increased with age, while sitting time decreased. Participants who practiced a combination of both outdoor and at-home workouts had higher total, housework and leisure-related PA, and higher moderate and vigorous-intensity PA. Machine learning models confirmed these findings as well as the importance of outdoor activity for total PA levels, with Random Forest being the highest-performing model. Conclusions: Bringing to light the levels of physical inactivity and sedentary behavior, this study highlighted the importance of outdoor activity in contributing to PA.

## 1. Introduction

The COVID-19 pandemic emerged in 2019 and has been since affecting the population worldwide, reaching more than 767 million cases by May of 2023 [1]. In Lebanon, the first case of COVID-19 was reported on 21 February 2020 [2], with the total number of cases reaching 1,237,556 to date [1]. In addition to its short-term health impacts, the COVID-19 pandemic has been associated with a number of dreadful long-term health consequences, namely, respiratory problems, and specifically, chronic obstructive pulmonary disease (COPD) [3]. In particular, this has also had an unfortunate impact on unvaccinated elite athletes; in fact, COVID-19 infection was shown to affect respiratory muscle strength and pulmonary function [4].

In the midst of the abovementioned threats, most countries imposed lockdowns as a measure to reduce the spread of the COVID-19 virus [5]. Although effective, this measure has shown a number of deleterious effects that ought to be taken into consideration, namely, metabolic, psychological, and economic effects [5]. Among these, one major aspect is the change in physical activity (PA) behavior, particularly physical inactivity. This is an essential aspect to consider, given that among COVID-19 patients, physical inactivity has been shown to increase the risks of hospitalization, admission to the intensive care unit, and death [6]. Moreover, increased PA has been shown to improve wellbeing and satisfaction during lockdowns [7]. Recent studies assessing the impact of lockdown on PA levels reported a decrease during lockdown in comparison to pre-lockdown levels, both worldwide [8,9,10,11,12,13] and in the Middle East and North Africa (MENA) region [14,15,16,17]. An increase in sedentary behavior and sitting time was also observed [14,18]. However, a study among Italians residing in the northwestern region of the country showed a moderate level of PA during a nationwide-imposed lockdown [19]. Although some of the studies conducted during the COVID-19 period included Lebanon [14,17], there is a lack of data extensively assessing PA during lockdown in the country. A recent study on eating behavior and confinement stressors conducted among 407 Lebanese adults showed that 41.1% of the participants were not practicing PA during confinement [20]. Another more recent study assessing dietary habits and lifestyle among 2507 Lebanese adults during lockdown reported that there was a significant decrease in PA levels as compared to pre-lockdown levels, as well as an increase in sedentary behavior [21]. Moreover, university students with daily smartphone screen time use equivalent to 7 h or higher during lockdown reported lower rates of PA than did their counterparts [22]. 

Increasing awareness of physical inactivity and its underlying predictors is of major importance, since PA has been proven to be effective in overcoming the challenges of the lockdown period, according to recent observations [23]. Moreover, evaluating the behaviors of specific subgroups leads to the development of more efficient interventions in the future. In order to achieve this, accurate and precise deductions are highly desirable in data analysis. For this purpose, recent technological advances have induced the use of machine learning and artificial intelligence in various domains [24]; the use of machine learning in PA certainly constitutes one of these advances [25,26]. We hypothesized that, through this study, we would be able to discern specific patterns, characteristics, and subgroups of the population through machine learning, allowing us to better shape future policies targeting PA levels. Thus, the objective of the current study is to assess PA during lockdown using the “International Physical Activity Questionnaire” (IPAQ)—long form, and evaluate its potential predictors, such as age, sex, work location, and type of PA. These predictors are then used for the building of models and are assessed through both classical statistics and machine learning, in order to obtain a better sense of the findings and derive more accurate conclusions regarding the predictors of PA levels.

## 2. Materials and Methods

### 2.1. Participants

The total sample included 795 participants, of which 218 were men (27.42%) and 577 were women (72.58%). Only 9.3% of our sample continued to work from the workplace (probably due to their being excluded from the lockdown restrictions), while the rest were either unemployed (45.3%) or working from home (45.3%). 

### 2.2. Study Design 

This is a cross-sectional study. To be included, participants had to be Lebanese, and aged between 18 and 64 years. The sampling type was a “snowball sampling”. The minimum sample size was calculated using the G-Power software, version 3.0.10. The calculated effect size was 0.0526, with an expected squared multiple correlation of 0.05 (R^2^ deviation from 0) related to the omnibus test of multiple regression. The minimum necessary sample was n = 371, considering an alpha error of 5%, a power of 80%, and allowing 15 predictors to be included in the model. A minimum sample of 400 participants would have to be targeted to take potential missing values into account; the targeted sample was doubled to account for the snowball technique’s clustering effect. Data was collected remotely, using an online questionnaire prepared through Google Forms. The link to the questionnaire was transmitted and shared via social media to reach the maximum number of people during the total lockdown period imposed by the Lebanese government from 7 January till 8 February 2021. Both the French and the Arabic versions of the questionnaire were available online from 26 January until 8 February 2021. Informed consent was obtained from the participants after explaining the study’s objectives. Only after consenting to the terms of participation were they able to advance and complete the survey. Their participation was voluntary, and information was kept anonymous. In order to account for double-communication bias, responses were checked for duplicated answers, as well as for repetitive submissions, the latter by means of the monitoring of the timestamps. 

The long form of the IPAQ was used to assess PA during lockdown. Either the validated French version of the IPAQ [27] or the previously validated Arabic IPAQ [28] were filled out by the participants. Other questions, which related to sociodemographic characteristics, weight and height, work location, PA change, and PA behaviors during lockdown, were also included in the form. The crowding index was defined as the total number of co-residents per household, divided by the total number of rooms, excluding the kitchen and the bathrooms; a crowding index value <1 indicates a good socioeconomic status. Questions related to the total number of co-residents per household, and to the total number of rooms, were also included in the sociodemographic questionnaire. 

The IPAQ assesses PA during the last seven days across four domains (occupation, transportation, housework, and leisure), in addition to sitting time during weekdays and the weekend. The long form of the IPAQ was used, because it contains questions about detailed intensity levels of PA in each of the four domains; it also includes questions about sitting time addressing both weekdays and weekend days. These parts are not present in the short-form IPAQ, and are important in gaining extra insights and better exploration of the population. Data obtained from the IPAQ was evaluated using the IPAQ scoring system [29], through the calculation of metabolic equivalents of PA domains (MET·min/week), and classifying PA according to a cut-off of 600 MET·min/week. This cut-off reflects the PA recommendations established by the Centers for Disease Control and Prevention, the American College of Sports Medicine, and the U.S. Department of Health and Human Services (30 min five times a week, hence reaching 150 min/week) [29]. 

### 2.3. Ethical Considerations 

The procedures of this study were aligned with the ethical standards of the institutional research committee as well as with the 1964 Helsinki Declaration and its amendments. The Ethics Committee of Saint Joseph University of Beirut (USJ) approved this study: approval was received 21 January 2021 for project “USJ–2021–21”. 

### 2.4. Statistical Analysis

Categorical variables were presented as frequency and percentages, and continuous variables as mean and standard deviation. A chi-square test was performed to evaluate the association between independent variables (age, sex, job location, and type of PA during lockdown), and the dependent variables (PA level and intensity, as well as sitting time). One-way ANOVA was performed to present the data as mean ± SD; nevertheless, results were considered as hypothesis-generating only, as the non-normality of the variables precluded drawing proper conclusions. The Spearman correlation coefficient was used to evaluate the correlation between age and PA domains. Multivariate logistic regression was performed to test the association between PA domains, PA intensity, and sitting time as dependent variables, and the independent variables (age, sex, work location, BMI, and crowding index). *p* < 0.05 was considered significant. Statistical analyses were performed using IBM SPSS (IBM SPSS Statistics for Windows, Version 20, IBM Corp., Armonk, NY, USA). 

### 2.5. Machine Learning Analysis

Different machine learning models were applied, with the target variable being a total PA higher or lower than 600 MET·min/week. It is thus a classification problem. Features included in the model involved age categories, sex, BMI, crowding index, education, marital status, job location during lockdown, auto-declared PA change during lockdown, type of PA during lockdown, employment status, and sitting time. In data preprocessing, since the features are categorical (object), “label encoding” was performed for age, sex, BMI, marital status, crowding index, education and sitting time, while “one hot encoding” was performed for employment status, job location during lockdown, PA change during lockdown, and type of PA during lockdown. The machine learning models that were compared included Random Forest, XGBoost, and SVM, in addition to neural networks. After splitting the sample into “train” and “test” sets, grid search was performed for each model before choosing the best parameters for each one of them. Metrics used to evaluate the models were retrieved, and included accuracy, precision, recall, and F1-score. SHAP analysis (Shapley additive explanations) was performed to visually represent the highest performing model; its use helps to improve the interpretability of the model, as well as explain the reasoning behind its predictions [30,31,32]. Analysis was performed using Python version 3.9.16, using Pandas version 1.5.3, Numpy version 1.22.4, Scikit-learn (sklearn) version 1.2.2, and SHAP version 0.41.0 libraries.

## 3. Results

### 3.1. Description of the Study Sample

The majority (67.5%) of the sample auto-declared that their PA levels decreased during the lockdown, as compared to their usual levels. One-third (34.2%) of the sample declared themselves as not practicing any PA, 24.3% practiced at-home workouts (online live sessions or pre-recorded), 24.0% chose outdoor activities, and 17.5% opted for a combination of at-home and outdoor activities. While 38.2% of our sample had a moderate PA level (600–3000 MET·min/week), and 25.5% had a high PA level (≥3000 MET·min/week), 36.2% did not meet the minimum recommendations for PA and were considered inactive (<600 MET·min/week). The results are presented in Table 1. 

### 3.2. PA Levels and Their Association with Participants’ Characteristics 

#### 3.2.1. PA and Sex

Table S1 (in the Supplementary Materials)  shows the association between PA, both as to level and intensity, and sex. While women had a significantly higher level of housework-related PA (*p* = 0.002), men had a higher job-related PA (*p* = 0.048) and a higher sitting time (*p* = 0.046).

#### 3.2.2. PA and Age

Table S2 shows the associations between age and PA level and intensity, in addition to sitting time. Figure S1 (Supplementary Materials) represents the results for total and leisure PA. Significantly higher PA levels were observed among participants aged ≥40 years in nearly all PA domains (total PA, transportation-related and housework-related PA, leisure PA, walking, and vigorous PA). Job-related PA was only the highest domain among participants aged 23–40 years. As for sitting time, it was at its highest among young participants aged <23 years. These results were further confirmed by the one-way ANOVA analysis of the mean PA levels. The Spearman correlation coefficient also showed a significant positive correlation between age and almost all PA domains assessed by the IPAQ, and a negative correlation with sitting time (see Table S3 in the Supplementary Materials). 

#### 3.2.3. PA and Work Location

Table 2 shows the association between PA, both as to level and intensity, and work location during lockdown. Participants who went to their workplace during lockdown had significantly higher proportions of total PA level, job-related PA, and walking PA, as well as moderate PA levels ≥600 MET·min/week, as compared to their counterparts who either worked from home or who were then unemployed. In addition, only 7.5% of the participants who continued to go to their workplaces were in the highest tertile for average sitting time per day (>10 h/day), and the majority (52.2%) sat less than 6.29 h/day. Figure S2 in the Supplementary Materials also represents the categories of total and walking-related PA (≥ and <600 MET·min/week) according to work location during lockdown. The analysis assessing the mean PA levels further confirmed these findings. 

A more detailed analysis of this association, as stratified by overweight status, is represented in Tables S4 and S5; these tables show the associations between PA level and intensity and work location during lockdown among two subgroups of overweight-status participants—BMI < 25 and ≥25 Kg/m^2^. An interesting finding is that, while the subgroup of BMI < 25 kept all the statistically significant associations observed previously among the total sample (total PA, job-related PA, walking PA, moderate PA, and sitting time), the statistically significant associations observed within the subgroup BMI ≥ 25 concerned only job-related PA, moderate PA, and sitting time, with the associations with total PA and walking becoming statistically insignificant.

#### 3.2.4. PA and BMI

Table S6 in the Supplementary Materials shows PA levels and intensity across BMI categories. No statistically significant differences were observed.

#### 3.2.5. PA Level and Type of PA 

Table S7 and Figure S3 in the Supplementary Materials show the associations between PA level and intensity and the type of PA practiced during lockdown. Participants who were practicing a combination of both outdoor and online activities at home had a significantly higher proportion of PA levels ≥ 600 MET·min/week, specifically, in total PA, housework-related, and leisure PA, than did their counterparts who either practiced outdoor activities only, or followed online programs, or did not practice any PA. Similar results were obtained with moderate and vigorous-intensity PA, while a higher proportion for walking PA was observed among those who practiced outdoor PA only. In contrast, those who did not practice any PA had the highest proportion of elevated sitting time (>10 h/day). These findings were further confirmed by the one-way ANOVA analysis of mean PA levels and sitting time.

Moreover, the majority (36.9%) of participants aged ≥40 years practiced mostly outdoor activities (*p* < 0.001), while the majority (34.8%) of younger participants did not practice any PA (Table S8). 

#### 3.2.6. Multivariate Analysis 

Multivariate analysis showed that PA level and intensity during lockdown (as measured by IPAQ) increased with age, with adjustment for sex, work location, BMI, and crowding index. On the contrary, sitting time decreased with age. Results also showed that total PA, as well as job-related, walking, and moderate PA levels were significantly higher among participants who worked from the office during lockdown, as compared to those who worked from home. Participants who went to the office also had less sitting time. Regarding sex, BMI, and the crowding index, they had no significant impact on the association with PA level and intensity. Nevertheless, women had a significantly lower sitting time than did men. The results are presented in Table 3.

### 3.3. Analysis Using Machine Learning Algorithms

**Figure 1:**
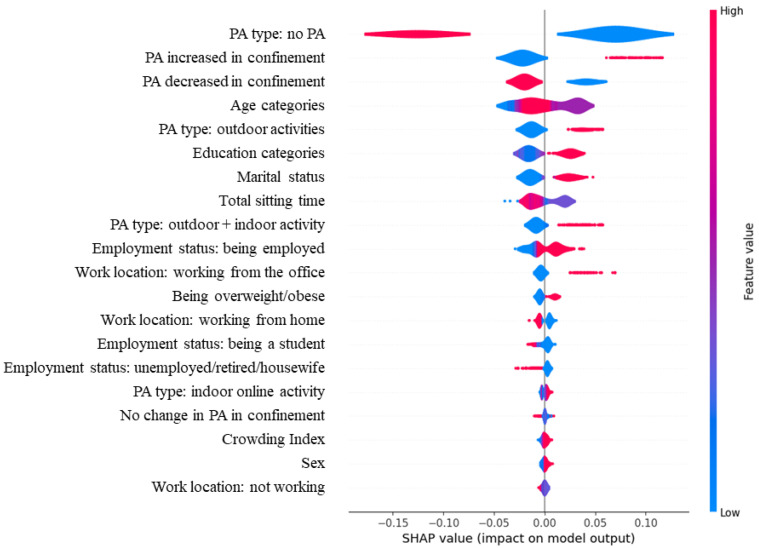
This figure is a SHAP analysis representation of the best-performing Random Forest model, showing the influence of the features on the total model, and thus on the target variable, which is here total PA ≥ 600 MET·min/week. The SHAP value shows the positive or negative impact of each feature on the model output (here, predicting total PA ≥ 600 MET·min/week). The feature value uses colors to represent the value of each feature, with red being a higher value, and blue being a lower one.

Table 4 represents the comparison of all models, showing accuracy, precision, recall and F1-score as metrics. The highest performing model was the Random Forest classifier. This model was obtained with the following parameters: criterion = ‘gini’, max_depth = 4, n_estimators = 200, and max_features = ‘auto’. Figure 1 is a SHAP analysis representation of this model showing the influence of the features on the total model, and thus on the target variable, which is here a total PA higher or lower than 600 MET·min/week. SHAP analysis allowed us to illustrate the different predictors of a PA level ≥ 600 MET·min/week, representing the positive or negative influence of each feature or variable (SHAP value) on the total model’s prediction of a higher total PA level. While it was expected that the results would demonstrate a high contribution to an increased total PA level from variables reflecting the practice of PA or an increase in PA level post-confinement (PA increased in confinement, not practicing PA), this analysis shed a light on the decreased PA level among younger, single participants, as compared to older, married participants. Participants with a higher educational level also tended to have a higher PA level. This analysis also shed light on a specific interesting effect, which was the impact of practicing outdoor PA on increased PA level, an effect that was present when outdoor PA was combined with online activities, but which, however, disappeared with indoor online activity alone. Moreover, working from the office had particularly a positive impact on increasing total PA level, while this was not present for working from home. 

Regarding XGBoost, the best parameters after the grid search were ‘learning_rate’ = 0.001, ‘max_depth’ = 3, and ‘n_estimators’ = 50. As for SVM, the best parameters were C = 1, degree = 2, and kernel = ‘poly’. A neural network model was performed, which included two dense layers—the first dense layer had 64 units with a rectified linear unit (ReLU) activation function, and the second dense layer had one unit with a sigmoid activation function. The parameters used in the neural network model were: the loss = ‘binary_crossentropy’, and the optimizer = ‘adam’. It was trained for seven epochs, with batch size = 32. Table 5 shows the results of the application of the above-mentioned models specifically for class 1, which is a total PA ≥ 600 MET·min/week.

Given that the model predicted class 1 (total PA ≥ 600 MET·min/week) better than it did class 0 (total PA < 600 MET·min/week), the analysis was repeated with the introduction of the class = ‘balanced’ or class = ‘balanced_subsample’ parameter to provide equal weights to both classes. The resulting metrics were hence improved for class 0. The corresponding metrics for the model and for class 1 are shown in Table 6 and Table 7, respectively. 

**Figure 2:**
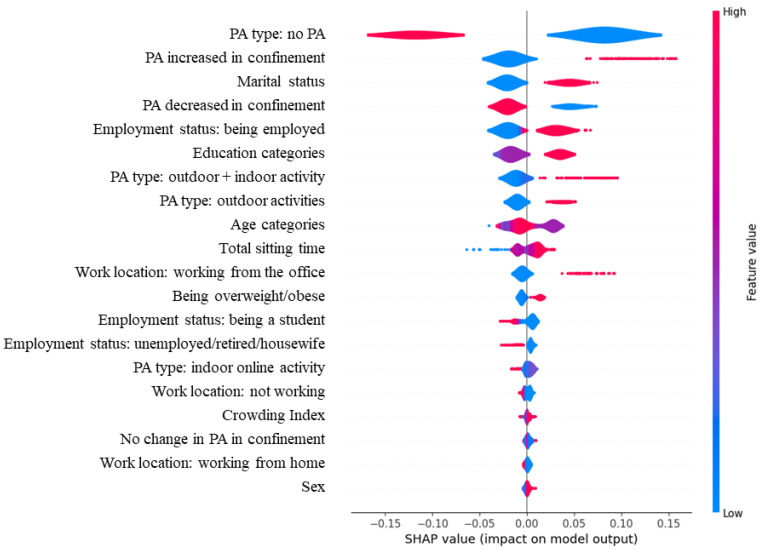
This figure is a SHAP analysis representation of the best-performing Random Forest model, with the use of balance weights (class_weight = balanced_subsample) showing the influence of the features on the total model, and thus on the target variable, which is here total PA ≥ 600 MET·min/week. The SHAP value shows the positive or negative impact of each feature on the model output (here predicting total PA ≥ 600 MET·min/week). The feature value uses colors to represent the value of each feature, with red being a higher value, and blue being a lower one.

Figure 2 shows a SHAP analysis representation of the Random Forest model, using balance weights. 

## 4. Discussion

To our knowledge, this is the first study to provide insights on PA behavior during the COVID-19 lockdown, using the IPAQ long form, among Lebanese adults. Around 36.2% of our sample did not meet the minimum recommendations for total PA and were considered inactive (<600 MET·min/week). Moreover, 62.5 to 71.1% had a PA level < 600 MET·min/week for leisure PA, total walking, and moderate and vigorous PA, in addition to a high sitting time. Our results showed that participants who worked from their offices during lockdown showed higher levels of PA and less sitting time. In addition, those who practiced PA both outdoors and at home scored higher PA levels. This was confirmed by the machine learning prediction model, which further exhibited the importance of outdoor activity for total PA levels. 

In accordance with recent studies worldwide [8,9,10,11,12,13], the majority of our sample reported a decrease in PA during lockdown, across all age and sex categories. These findings were also in line with studies conducted in Lebanon which reported an increase in sedentary behavior during lockdown [20,21]. Nevertheless, although the majority (66.9%) of our sample experienced a decrease in PA, around 16% increased their PA. This could be explained by many reasons. Previous findings have shown an increase in leisure-related PA during lockdown, specifically, among individuals already involved in sports training, such as bodyweight training, and who gained more time to exercise at home [9,11]. Moreover, a previous study by Maugeri et al. suggested that subjects who were less active before lockdown significantly increased their PA due to their having more flexible schedules and more free time [13]. In another study conducted in Saudi Arabia, those who exercised with a personal trainer kept the same PA level during lockdown, as compared to pre-lockdown levels [33]. A recent review also highlighted the increase in PA among specific population subgroups, such as undergraduate students who practiced cycling and running during the pandemic. It also emphasized the role and the positive influence of the outdoor neighboring environments on maintaining an active lifestyle, namely backyards, sidewalks, parks and trails and other spaces of the built environment [34]. 

In our study, participants who practiced both outdoor and at-home activities were the most active (PA ≥ 600 MET·min/week), followed by participants who only practiced outdoor activities such as walking, jogging, and hiking. These findings were highlighted by both multivariate regression analysis and machine learning prediction models. In fact, the results of the SHAP analysis presented the importance of outdoor activities in achieving a total PA level ≥ 600 MET·min/week. It also revealed the role of working from the office in maintaining and achieving a higher PA level. In a study conducted among adults in the United States on PA locations and behaviors, while “more vigorous and moderate activities were reported by those who were active in their home/garage or driveway/yard, more steps were recorded by those who were active on the roads in their neighborhood, or who used parks/trails” [35]. This highlights the importance of preserving outdoor activities in everyday life to increase PA levels. All of these findings showcase some good alternatives to gyms and fitness centers for staying physically active during the pandemic. A particular finding obtained in the study is the more pronounced association between working from the office and PA level among participants having a BMI < 25 kg/m^2^, as compared to overweight/obese participants; this, interestingly, concerned walking PA. As a matter of fact, a higher BMI was associated with a lower PA level in many studies conducted during COVID-19 [36]. The results of the current study either point to the behavior of people with higher BMI, in terms of avoiding walking and opting for other PA activities, if any, or to the preferences of participants with lower BMI, in terms of being more inclined to exercise. In all cases, despite the spread of COVID-19 and the lockdown measures, it is important to have stayed physically active, and to have aimed for 150 min per week of moderate-intensity PA, as recommended by the CDC [37] and the WHO [38], while maintaining social distancing when opting for outside activities [37,38]. It has been previously shown that even a minor decrease in PA can have a deleterious impact on both physical and mental health [5]. Therefore, aiming for even incremental increases in PA can induce several benefits and improvements in one’s health [5]. 

Aside from the decrease in PA, there was a high prevalence of time spent sitting, probably in front of screens, whether it was for work, study, or both, or for entertainment purposes. Recent findings in the MENA region reported that 35% of participants spent more than five hours per day facing screens [14]. Likewise, according to recent findings from Lebanon, 34.8% spent more than 5 h in front of the screen for study or work purposes compared to 19.9% pre-lockdown, while 38.8% spent more than 5 h in front of screens for entertainment purposes, compared to 11.2% pre-lockdown [21]. In contrast, we observed that participants who worked from the office achieved higher PA levels and lower time spent sitting, as compared to their counterparts who stayed at home. This was in line with a recent study showing that employees who worked online from home were less physically active and reached a higher level of sedentary lifestyle, as compared to their counterparts in workplaces [39]. Too much sitting has been associated with numerous adverse health effects, including cardiovascular diseases, diabetes, a higher mortality risk, as well as deteriorated mental health; these associations remained significant even after accounting for time spent in PA [40,41]. The above-mentioned findings shed a light on the importance of establishing adequate social and work-related policies aiming to balance the benefits of both remote work and outdoor activities. 

Finally, the results of the statistical analysis of this study showed that PA level and intensity during lockdown increased with age, after adjusting for sex, work location, BMI, and crowding index, whereas sitting time decreased with age. This might be explained by the fact that the younger subjects included in our study were students, so they were probably spending most of their time during weekdays following their university courses online, in addition to spending more screen time on entertainment purposes, and hence becoming less active. Conversely, older adults probably had more free time to spend doing less sedentary activities during the lockdown period. Findings from machine learning and SHAP analysis confirmed the trend observed among younger participants. As for the older age categories, the findings were less consistent, given that the contribution of the age factor to the whole model was mostly predominant among participants aged 24–39 years. The trend that we observed as being associated with age was not evident in other studies, despite some findings about an increase in leisure-related PA during lockdown among people who were already involved in training, and who gained more time to exercise at home [9,11]. Further studies are needed to investigate PA and lifestyle behaviors among older age groups during lockdowns. No significant differences were observed among sex categories concerning the PA change during lockdown in our study, except for a higher sitting time found among men. This was confirmed by SHAP analysis, where sex remained in the lowest levels of contribution to the whole model’s prediction of a total PA ≥ 600 MET·min/week. Accordingly, only a few studies have previously reported sex differences, specifically, a larger decrease of PA among men during lockdown, as compared to pre-lockdown activity [10,11,13]. Regarding BMI categories, no statistically significant differences were observed. In SHAP analysis, the effect of being overweight/obese was not impactful; however, it was in the middle of potential factors relative to the features’ importance level. With many studies in the literature showing that a higher BMI was constantly associated with a lower PA level [36], many reasons could explain the insignificance of the association found here, namely the cross-sectional design of the study, and the limited number of participants in each of the BMI categories to allow for more detailed analysis. 

The strengths of this study include the use of the long-form IPAQ, which allows the assessment of many aspects of PA. Moreover, the use of Google Forms helped in reaching a large number of people with an easily-accessible, cost-effective, and time-saving method, while respecting confinement and social distancing. Regarding the analytical aspects of the machine-learning model, its performance was “decent” enough to support the findings of this study, but not enough be used alone to predict total PA. This is completely understandable in machine learning, where data itself is the foundation and the main influence on analysis. Nevertheless, the findings still offered important insights to be considered in future lockdowns, as online programs ultimately could not replace outdoor PA. In addition, the use of machine-learning models provided more precision and accuracy for our results, and the use of SHAP analysis allowed for a better interpretability of the study’s findings. 

A limitation of this study is the self-reported questionnaire, which reflects possible misreporting bias. Another limitation is the short time-interval of the study; longitudinal studies could provide more insights in this matter. Moreover, future studies should thoroughly assess human behavior during lockdown, as they would allow the development of adequate recommendations for the public. 

## 5. Conclusions

This study showed a trend of sedentary behavior and physical inactivity during COVID-19-induced lockdown, although some age groups opted to increase their PA in their free time. In the future, particular focus should be placed on lifestyle recommendations related to remote work, in order to counterbalance the negative effects of this emerging new reality. Developing effective alternatives to gyms/fitness centers is of particular importance, especially among individuals overwhelmed by busy schedules. The study’s findings shed a light on the importance of outdoor activity as part of total PA, given that this type of PA still could not be fully replaced by indoor/online programs. One specific tool worth being further examined in this context is “exergaming”; although it has its pitfalls, it might have a notable influence on PA level [42]. That said, the optimal balance between outdoor and indoor activities should be better-planned in future lockdowns. 

## Figures and Tables

**Table 1:** Description of the study sample.

	Total Sample (n = 795)	Men (n = 218)	Women (n = 577)	*p*-Value *
Age (mean ± SD)	33.13 ± 14.39	33.24 ± 16.0	33.07 ± 13.71	0.891 ^†^
Age n (%)				0.096 *
<23 years	302 (38.0)	95 (43.6)	207 (35.9)	
24–39 years	241 (30.3)	56 (25.7)	185 (32.1)	
≥40 years	252 (31.7)	67 (30.7)	185 (32.1)	
Crowding index n (%)				0.563 *
<1	461 (58.0)	130 (59.6)	331 (57.4)	
≥1	334 (42.0)	88 (40.4)	246 (42.6)	
Marital status n (%)				0.727 *
Single	489 (61.5)	138 (63.3)	351 (60.8)	
Engaged/Married	279 (35.1)	74 (33.9)	205 (35.5)	
Divorced/Widowed	27 (3.4)	6 (2.8)	21 (3.6)	
Education n (%)				0.237 *
High school or less	248 (31.2)	77 (35.3)	171 (29.6)	
Bachelor	247 (31.1)	60 (27.5)	187 (32.4)	
Master’s degree or above	300 (37.7)	81 (37.2)	219 (38.0)	
BMI (kg/m^2^) (n = 793) n (%)				<0.001 *
<18.5	62 (7.8)	9 (4.1)	53 (9.2)	
18.5–24.9	462 (58.1)	95 (43.8)	367 (63.7)	
25–29.9	208 (26.2)	91 (41.9)	117 (20.3)	
≥30	61 (7.7)	22 (10.1)	39 (6.8)	
Employment category n (%)				0.001 *
Employed	370 (46.5)	108 (49.5)	262 (45.4)	
Student	268 (33.7)	85 (39.0)	183 (31.7)	
Unemployed/Retired/Housewife	157 (19.7)	25 (11.5)	132 (22.9)	
Job location during lockdown n (%)				0.006 *
Home	360 (45.3)	94 (43.1)	266 (46.1)	
Office	74 (9.3)	32 (14.7)	42 (7.3)	
Student/Unemployed	361 (45.4)	92 (42.2)	269 (46.6)	
Auto-declared PA change during lockdown n (%)				0.405 *
Did not change	130 (16.4)	38 (17.4)	92 (15.9)	
Decreased	537 (67.5)	151 (69.3)	386 (66.9)	
Increased	128 (16.1)	29 (13.3)	99 (17.2)	
Type of PA during lockdown n (%)				0.542 *
At-home workouts	193 (24.3)	48 (22.0)	145 (25.1)	
Outdoor activities (walking, jogging, hiking)	191 (24.0)	51 (23.4)	140 (24.3)	
At-home and outdoor activities	139 (17.5)	36 (16.5)	103 (17.9)	
No PA	272 (34.2)	83 (38.1)	189 (32.8)	

BMI: body mass index; PA: physical activity; * Statistical Test: Chi-square test; ^†^: independent samples *t*-test; *p* < 0.05 was considered to be significant.

**Table 2:** Association between PA levels and intensity (as measured by IPAQ) and Work Location during lockdown.

	Home (n = 360)	Office (n = 74)	Student/Unemployed (n = 361)	*p*-Value
Total PA level n (%)				<0.001
<600 MET·min/week	131 (36.4)	10 (13.5)	147 (40.7)	
≥600 MET·min/week	229 (63.6)	64 (86.5)	214 (59.3)	
Job-related PA n (%)				<0.001
<600 MET·min/week	327 (90.8)	29 (39.2)	354 (98.1)	
≥600 MET·min/week	33 (9.2)	45 (60.8)	7 (1.9)	
Transportation-related PA n (%)				0.093
<600 MET·min/week	322 (89.4)	64 (86.5)	303 (83.9)	
≥600 MET·min/week	38 (10.6)	10 (13.5)	58 (16.1)	
Housework-related PA n (%)				0.936
<600 MET·min/week	234 (65.0)	48 (64.9)	239 (66.2)	
≥600 MET·min/week	126 (35.0)	26 (35.1)	122 (33.8)	
Leisure-related PA n (%)				0.061
<600 MET·min/week	237 (65.8)	42 (56.8)	254 (70.4)	
≥600 MET·min/week	123 (34.2)	32 (43.2)	107 (29.6)	
Walking n (%)				<0.001
<600 MET·min/week	259 (71.9)	32 (43.2)	257 (71.2)	
≥600 MET·min/week	101 (28.1)	42 (56.8)	104 (28.8)	
Moderate PA n (%)				<0.001
<600 MET·min/week	256 (71.1)	36 (48.6)	273 (75.6)	
≥600 MET·min/week	104 (28.9)	38 (51.4)	88 (24.4)	
Vigorous PA n (%)				0.093
<600 MET·min/week	225 (62.5)	38 (51.4)	234 (64.8)	
≥600 MET·min/week	135 (37.5)	36 (48.6)	127 (35.2)	
Sitting time (hours/day)				<0.001
Tertile 1 = ≤6.29 h	101 (29.7)	35 (52.2)	109 (33.7)	
Tertile 2 = 6.29–10 h	127 (37.4)	27 (40.3)	107 (33.1)	
Tertile 3 = >10 h	112 (32.9)	5 (7.5)	107 (33.1)	

*p* < 0.05 was considered to be significant; PA, physical activity; MET, metabolic equivalent task; Total PA level (MET-minutes/week) = Total Physical Activity MET-minutes/week = Sum of (Job PA + Transportation PA + Housework PA + Leisure PA MET-minutes/week scores).

**Table 3:** Multivariate analysis showing the association between PA level and intensity during lockdown (as measured by the IPAQ) and sociodemographic variables.

	≥600 vs. <600 MET·min/week ORs ^a^ (95% CI)
Total PA	
Age 23–40 vs. <23 y	1.220 (0.847–1.756)
Age ≥ 40 vs. <23 y	3.033 ^†^ (2.002–4.593)
Women vs. Men	1.078 (0.762–1.527)
Office vs. Home	3.688 ^†^ (1.802–7.547)
Job-related PA	
Age 23–40 vs. <23 y	5.252 ^†^ (2.099–13.138)
Age ≥ 40 vs. <23 y	3.977 ^†^ (1.531–10.332)
Women vs. Men	0.853 (0.460–1.581)
Office vs. Home	13.445 ^†^ (7.269–24.865)
Transportation-related PA	
Age 23–40 vs. <23 y	0.983(0.552–1.752)
Age ≥ 40 vs. <23 y	2.430 ^†^ (1.423–4.149)
Women vs. Men	0.691 (0.433–1.103)
Office vs. Home	1.223 (0.568–2.632)
Housework-related PA	
Age 23–40 vs. <23 y	0.923 (0.624–1.365)
Age ≥ 40 vs. <23 y	1.928 ^†^ (1.305–2.848)
Women vs. Men	1.745 (1.215–2.505)
Office vs. Home	1.085 (0.630–1.868)
Leisure-related PA	
Age 23–40 vs. <23 y	1.040 (0.704–1.538)
Age ≥ 40 vs. <23 y	1.798 * (1.209–2.675)
Women vs. Men	0.783 (0.555–1.105)
Office vs. Home	1.417 (0.841–2.390)
Walking	
Age 23–40 vs. <23 y	1.591 * (1.057–2.395)
Age ≥ 40 vs. <23 y	2.733 ^†^ (1.800–4.149)
Women vs. Men	0.943 (0.657–1.355)
Office vs. Home	3.236 ^†^ (1.904–5.500)
Moderate PA	
Age 23–40 vs. <23 y	1.107 (0.738–1.660)
Age ≥ 40 vs. <23 y	1.424 (0.941–2.154)
Women vs. Men	0.835 (0.585–1.193)
Office vs. Home	2.452 ^†^ (1.457–4.126)
Vigorous PA	
Age 23–40 vs.<23 y	1.235 (0.843–1.809)
Age ≥ 40 vs.<23 y	2.470 ^†^ (1.672–3.649)
Women vs. Men	1.286 (0.909–1.820)
Office vs. Home	1.592 (0.945–2.681)
Sitting time	Tertile 3 vs. Tertile 1
Age 23–40 vs.<23 y	0.615 (0.376–1.007)
Age ≥ 40 vs.<23 y	0.220 ^†^ (0.132–0.367)
Women vs. Men	0.612 * (0.388–0.967)
Office vs. Home	0.120 ^†^ (0.044–0.327)
Sitting time	Tertile 2 vs. Tertile 1
Age 23–40 vs.<23 y	0.701 (0.434–1.134)
Age ≥ 40 vs.<23 y	0.334 ^†^ (0.206–0.541)
Women vs. Men	0.570 * (0.371–0.877)
Office vs. Home	0.564 (0.310–1.024)

^a^ Further adjusted for body mass index, and crowding index; ^†^ *p* < 0.01, * *p* < 0.05, *p* < 0.05 was considered to be significant. Crowding index was defined as the total number of co-residents per household, divided by the total number of rooms, excluding the kitchen and the bathrooms; PA: physical activity; OR: odds ratio; CI: confidence interval; MET·min/week: metabolic equivalent per week; y: years.

**Table 4:** Comparison of performance metrics of different machine learning models.

	Accuracy	Precision	Recall	F1-Score
Train set				
Random Forest	0.74	0.82	0.74	0.76
XGBoost	0.73	0.76	0.73	0.74
SVM	0.76	0.79	0.76	0.77
Neural Network	0.76	0.76	0.76	0.75
Test set				
Random Forest	0.75	0.82	0.75	0.77
XGBoost	0.75	0.76	0.75	0.76
SVM	0.68	0.69	0.68	0.69
Neural Network	0.71	0.71	0.71	0.70

Weighted average results; target variable: total PA higher or lower than 600 MET·min/week; features included in the model involved age categories, sex, BMI, crowding index, education, marital status, job location during lockdown, auto-declared PA change during lockdown, type of PA during lockdown, employment status, and sitting time.

**Table 5:** Comparison of performance metrics of different machine learning models (results of class 1).

	Accuracy	Precision	Recall	F1-Score
Train set				
Random Forest	0.74	0.93	0.73	0.82
XGBoost	0.73	0.86	0.75	0.80
SVM	0.75	0.85	0.78	0.81
Neural Network	0.76	0.78	0.90	0.83
Test set				
Random Forest	0.75	0.92	0.76	0.83
XGBoost	0.75	0.84	0.81	0.82
SVM	0.68	0.78	0.74	0.76
Neural Network	0.71	0.72	0.84	0.77

Class 1 results; target variable: total PA higher or lower than 600 MET·min/week; features included in the model involved age categories, sex, BMI, crowding index, education, marital status, job location during lockdown, auto-declared PA change during lockdown, type of PA during lockdown, employment status, and sitting time.

**Table 6:** Comparison of performance metrics of different machine learning models after introducing the balanced parameter (giving more weight to class 0).

	Accuracy	Precision	Recall	F1-Score
Train set				
Random Forest	0.76	0.77	0.77	0.76
XGBoost	0.73	0.73	0.73	0.73
SVM	0.778	0.78	0.78	0.77
Neural Network	0.73	0.72	0.73	0.70
Test set				
Random Forest	0.76	0.76	0.76	0.76
XGBoost	0.67	0.67	0.67	0.67
SVM	0.69	0.69	0.69	0.69
Neural Network	0.65	0.64	0.65	0.62

Weighted average results; target variable: total PA higher or lower than 600 MET·min/week; features included in the model involved age categories, sex, BMI, crowding index, education, marital status, job location during lockdown, auto-declared PA change during lockdown, type of PA during lockdown, employment status, and sitting time.

**Table 7:** Comparison of performance metrics of different machine learning models after introducing the balanced parameter (giving more weight to class 0). (Results of class 1.)

	Accuracy	Precision	Recall	F1-Score
Train set				
Random Forest	0.76	0.76	0.81	0.81
XGBoost	0.73	0.76	0.82	0.79
SVM	0.74	0.82	0.78	0.80
Neural Network	0.73	0.73	0.92	0.81
Test set				
Random Forest	0.76	0.81	0.80	0.80
XGBoost	0.67	0.68	0.74	0.71
SVM	0.66	0.74	0.74	0.74
Neural Network	0.65	0.65	0.86	0.74

Class 1 results; target variable: total PA higher or lower than 600 MET·min/week; features included in the model involved age categories, sex, BMI, crowding index, education, marital status, job location during lockdown, auto-declared PA change during lockdown, type of PA during lockdown, employment status, and sitting time.

## Data Availability

The data presented in this study are available on request from the corresponding author. The data are not publicly available due to privacy reasons.

## Supplementary Materials

The following supporting information can be downloaded at: https://www.mdpi.com/article/10.3390/healthcare11142080/s1, Table S1: PA (IPAQ) characteristics according to Sex; Table S2: Association between Age and PA; Table S3: Spearman correlation coefficient between Age and PA domain, as assessed by the IPAQ; Figure S1: Categories of Total and Leisure PA (≥ and <600 MET·min/week) according to Age groups; Figure S2: Categories of Total and Walking-related PA (≥ and <600 MET·min/week) according to work location during lockdown; Table S4: Association between PA level and intensity (as measured by IPAQ) and Work Location during lockdown among participants with BMI < 25 kg/m^2^ (n = 524); Table S5: Association between PA level and intensity (as measured by IPAQ) and Work Location during lockdown among participants with BMI ≥ 25 kg/m^2^ (n = 269); Table S6: Association between PA level and intensity (as measured by IPAQ) and BMI categories during lockdown; Table S7: Association between PA level and intensity (as measured by IPAQ) and type of PA during lockdown; Figure S3: Categories of Total and Leisure PA (≥ and <600 MET·min/week) according to type of PA during lockdown; Table S8: Association between Age and type of PA during lockdown.
