# Supplementary material for: Physical Activity Levels and Predictors during COVID-19 Lockdown among Lebanese Adults: The Impacts of Sociodemographic Factors, Type of Physical Activity and Work Location
Source: Healthcare (Basel). 2023 Jul 21;11(14):2080. doi: 10.3390/healthcare11142080 (PMC10378799; doi:10.3390/healthcare11142080)
Supplement: Supplementary file 1 [file healthcare-11-02080-s001.zip › healthcare-2481469-supplementary.pdf]

Supplementary material

Physical Activity Levels and Predictors during COVID-19 Lockdown among Lebanese Adults: The Impacts of Sociodemographic Factors, Type of Physical Activity and Work Location

Table of content

**Content**	**Page**
**Table S1** PA (IPAQ) characteristics according to sex	S2
**Table S2** Association between age and PA	S4
**Table S3** Spearman correlation coefficient between age and PA domain assessed by the IPAQ	S6
**Figure S1** showing categories of Total and Leisure PA (≥ and <600 MET.min/week) according to age groups	S7
**Figure S2** showing categories of Total and walking-related PA (≥ and <600 MET.min/week) according to work location during lockdown	S8
**Table S4** Association between PA levels & intensity (as measured by IPAQ) and work location during lockdown among participants with BMI <25Kg/m2 (n=524)	S9
**Table S5** Association between PA levels & intensity (as measured by IPAQ) and work location during lockdown among participants with BMI ≥25Kg/m2 (n=269)	S10
**Table S6** Association between PA levels & intensity (as measured by IPAQ) and BMI categories during lockdown	S11-12
**Table S7** Association between PA levels & intensity (as measured by IPAQ) and type of PA during lockdown	S13-14
**Figure S3** Categories of total and leisure PA (≥ and <600 MET.min/week) according to type of PA during lockdown	S15
**Table S8** Association between age and type of PA during lockdown	S16

Table S1. PA (IPAQ) characteristics according to sex

	**Men** n = 218	**Women** n = 577	**Total** n=795	*p*-value *
Total PA level n (%)				0.865
<600 MET.min/week	80 (36.7)	208 (36.0)	288 (36.2)	
≥600 MET.min/week	138 (63.3)	369 (64.0)	507 (63.8)	
Job-related PA n (%)				0.048
<600 MET.min/week	187 (85.8)	523 (90.6)	710 (89.3)	
≥600 MET.min/week	31 (14.2)	54 (9.4)	85 (10.7)	
Transportation-related PA n (%)				0.249
<600 MET.min/week	184 (84.4)	505 (87.5)	689 (86.7)	
≥600 MET.min/week	34 (15.6)	72 (12.5)	106 (13.3)	
Housework-related PA n (%)				0.002
<600 MET.min/week	161 (73.9)	360 (62.4)	521 (65.5)	
≥600 MET.min/week	57 (26.1)	217 (37.6)	274 (34.5)	
Leisure-related PA n (%)				0.226
<600 MET.min/week	139 (63.8)	394 (68.3)	533 (67.0)	
≥600 MET.min/week	79 (36.2)	183 (31.7)	262 (33.0)	
Walking n (%)				0.697
<600 MET.min/week	148 (67.9)	400 (69.3)	548 (68.9)	
≥600 MET.min/week	70 (32.1)	177 (30.7)	247 (31.1)	
Moderate PA n (%)				0.164
<600 MET.min/week	147 (67.4)	418 (72.4)	565 (71.1)	
≥600 MET.min/week	71 (32.6)	159 (27.6)	230 (28.9)	
Vigorous PA n (%)				0.152

<600 MET.min/week	145 (66.5)	352 (61.0)	497 (62.5)	
≥600 MET.min/week	73 (33.5)	225 (39.0)	298 (37.5)	
Sitting time (hours/day)				0.046
Tertile 1 = ≤6.29 h	53 (26.6)	192 (36.2)	245 (30.8)	
Tertile 2 = 6.29-10 h	81 (40.7)	180 (33.9)	261 (32.8)	
Tertile 3 = >10 h	65 (32.7)	159 (29.9)	224 (28.2)	

*Statistical Test: Chi-square test; $p<0.05$ was considered as significant; PA, Physical activity; MET, metabolic equivalent task; Total PA level (MET-minutes/week) = Total physical activity MET-minutes/week = sum of (Job PA + Transportation PA + Housework PA + Leisure PA MET-minutes/week scores).

Table S2. Association between age and PA

	**<23 years**<br>**n = 302**	**23-40 years**<br>**n = 241**	**≥ 40 years**<br>**n=252**	***p*-value**<br>*****
Total PA level n (%)				<0.001
<600 MET.min/week	142 (47.0)	92 (38.2)	54 (21.4)	
≥600 MET.min/week	160 (53.0)	149 (61.8)	198 (78.6)	
Job-related PA n (%)				<0.001
<600 MET.min/week	295 (97.7)	197 (81.7)	218 (86.5)	
≥600 MET.min/week	7 (2.3)	44 (18.3)	34 (13.5)	
Transportation-related PA n (%)				0.004
<600 MET.min/week	267 (88.4)	218 (90.5)	204 (81.0)	
≥600 MET.min/week	35 (11.6)	23 (9.5)	48 (19.0)	
Housework-related PA n (%)				<0.001
<600 MET.min/week	213 (70.5)	171 (71.0)	137 (54.4)	
≥600 MET.min/week	89 (29.5)	70 (29.0)	115 (45.6)	
Leisure-related PA n (%)				0.008
<600 MET.min/week	215 (71.2)	168 (69.7)	150 (59.5)	
≥600 MET.min/week	87 (28.8)	73 (30.3)	102 (40.5)	
Walking n (%)				<0.001
<600 MET.min/week	237 (78.5)	163 (67.6)	148 (58.7)	
≥600 MET.min/week	65 (21.5)	78 (32.4)	104 (41.3)	
Moderate PA n (%)				0.057
<600 MET.min/week	228 (75.5)	170 (70.5)	167 (66.3)	
≥600 MET.min/week	74 (24.5)	71 (29.5)	85 (33.7)	

Vigorous PA n (%)				<0.001
<600 MET.min/week	214 (70.9)	156 (64.7)	127 (50.4)	
600-3000 MET.min/week	88 (29.1)	85 (35.3)	125 (49.6)	
Sitting time (hours/day)				<0.001
Tertile 1 = ≤6.29 h	56 (20.8)	68 (31.1)	121 (50.0)	
Tertile 2 = 6.29-10 h	105 (39.0)	84 (38.4)	72 (29.8)	
Tertile 3 = >10 h	108 (40.1)	67 (30.6)	49 (20.2)	

*Statistical Test: Chi-square test; $p<0.05$ was considered as significant; PA, Physical activity; MET, metabolic equivalent task; Total PA level (MET-minutes/week) = Total physical activity MET-minutes/week = sum of (Job PA + Transportation PA + Housework PA + Leisure PA MET-minutes/week scores).

Table S3 Spearman correlation coefficient between age and PA domain assessed by the IPAQ

	**r**	***p***
Total PA	0.212	<0.001
Job-related PA	0.203	<0.001
Transportation-related PA	0.110	0.002
Housework-related PA	0.122	0.001
Leisure-related PA	0.102	0.004
Walking	0.214	<0.001
Moderate PA	0.043	0.227
Vigorous PA	0.190	<0.001
Sitting time (hours/day)	-0.294	<0.001

r: Spearman correlation coefficient; $p<0.05$ was considered as significant; PA: physical activity

Figure S1 showing categories of Total and Leisure PA (≥ and <600 MET.min/week) according to age groups: participants aged ≥40 years showed the highest PA levels, $p<0.01$ for Total PA and Leisure PA

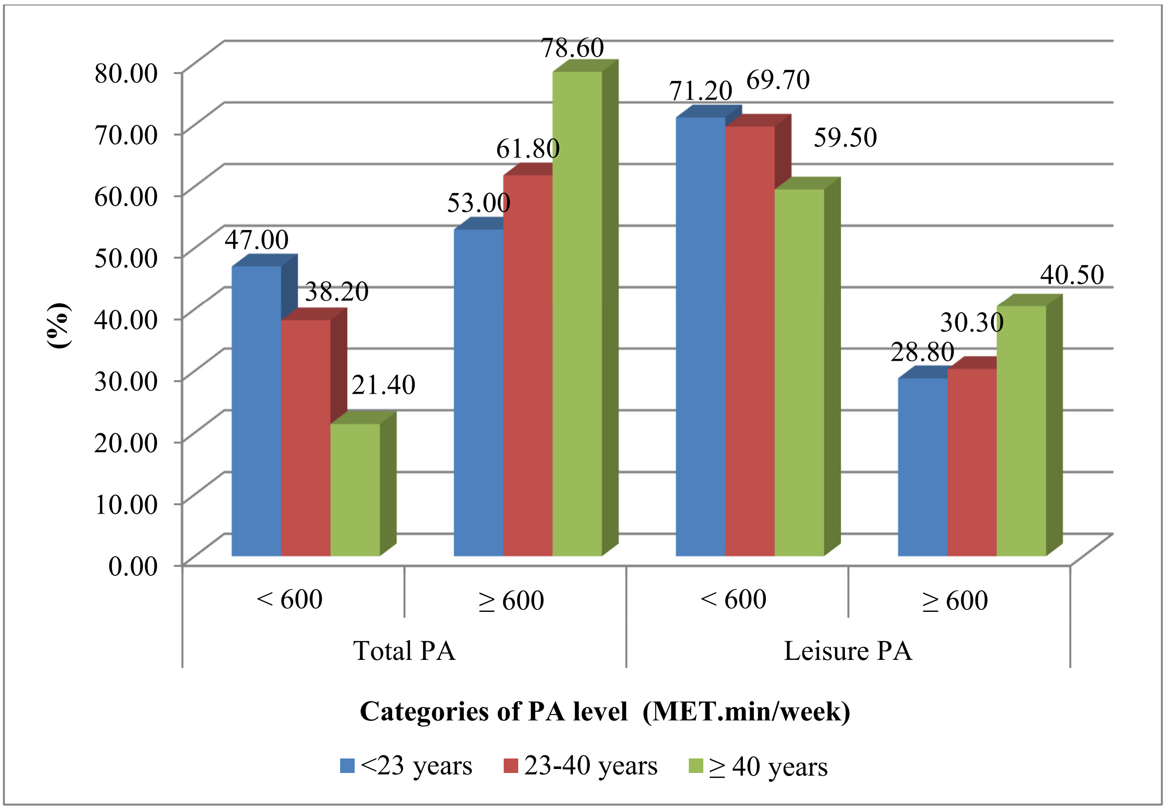

Figure S2 showing categories of Total and walking-related PA (≥ and <600 MET.min/week) according to work location during lockdown

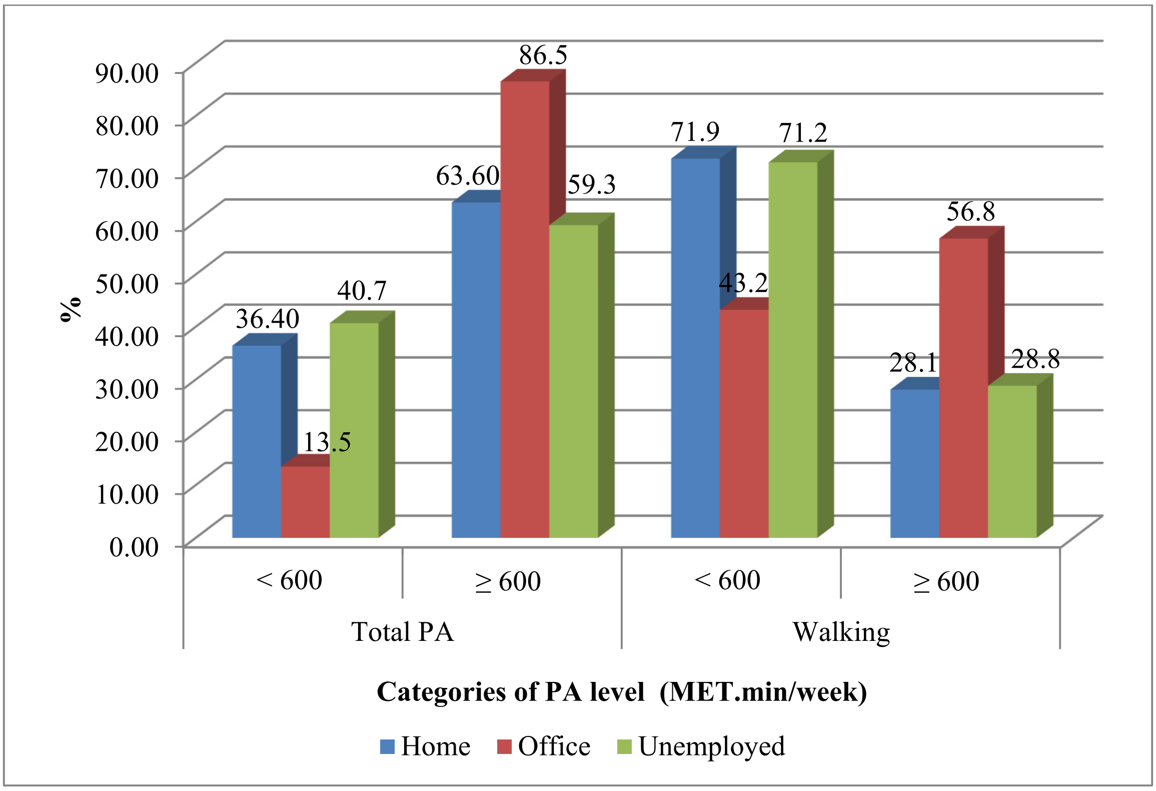

Table S4 Association between PA levels & intensity (as measured by IPAQ) and work location during lockdown among participants with BMI <25Kg/m[2] (n=524)

	**Home (n=237)**	**Office (n=45)**	**Student / Unemployed (n=242)**	***p*-value**
Total PA level n (%)				<0.001
<600 MET.min/week	91 (38.4)	5 (11.1)	103 (42.6)	
≥600 MET.min/week	146 (61.6)	40 (88.9)	139 (57.4)	
Job–related PA n (%)				<0.001
<600 MET.min/week	225 (94.9)	13 (28.9)	237 (97.9)	
≥600 MET.min/week	12 (5.1)	32 (71.1)	5 (2.1)	
Transportation–related PA n (%)				0.429
<600 MET.min/week	212 (89.5)	39 (86.7)	207 (85.5)	
≥600 MET.min/week	25 (10.5)	6 (13.3)	35 (14.5)	
Housework–related PA n (%)				0.989
<600 MET.min/week	160 (67.5)	30 (66.7)	164 (67.8)	
≥600 MET.min/week	77 (32.5)	15 (33.3)	78 (32.2)	
Leisure–related PA n (%)				0.124
<600 MET.min/week	155 (65.4)	26 (57.8)	173 (71.5)	
≥600 MET.min/week	82 (34.6)	19 (42.2)	69 (28.5)	
Walking n (%)				<0.001
<600 MET.min/week	174 (73.4)	13 (28.9)	178 (73.6)	
≥600 MET.min/week	63 (26.6)	32 (71.1)	64 (26.4)	
Moderate PA n (%)				0.001
<600 MET.min/week	175 (73.8)	22 (48.9)	181 (74.8)	
≥600 MET.min/week	62 (26.2)	23 (51.1)	61 (25.2)	
Vigorous PA n (%)				0.202
<600 MET.min/week	159 (67.1)	24 (53.3)	159 (65.7)	
≥600 MET.min/week	78 (32.9)	21 (46.7)	83 (34.3)	
Sitting time (hours/day)				0.002
Tertile 1 = ≤6.29 h	67 (29.4)	23 (53.5)	65 (30.5)	
Tertile 2 = 6.29-10 h	71 (31.1)	16 (37.2)	76 (35.7)	
Tertile 3 = >10 h	90 (39.5)	4 (9.3)	72 (33.8)	

$p<0.05$ was considered as significant; PA, Physical activity; MET, metabolic equivalent task; Total PA level (MET-minutes/week) = Total physical activity MET-minutes/week = sum of (Job PA + Transportation PA + Housework PA + Leisure PA MET-minutes/week scores).

Table S5 Association between PA levels & intensity (as measured by IPAQ) and work location during lockdown among participants with BMI ≥25Kg/m^2 (n=269)

	**Home (n=122)**	**Office (n=29)**	**Student / Unemployed (n=118)**	***p*-value**
Total PA level n (%)				0.142
<600 MET.min/week	40 (32.8)	5 (17.2)	43 (36.4)	
≥600 MET.min/week	82 (67.2)	24 (82.8)	75 (63.6)	
Job–related PA n (%)				<0.001
<600 MET.min/week	102 (83.6)	16 (55.2)	116 (98.3)	
≥600 MET.min/week	20 (16.4)	13 (44.8)	2 (1.7)	
Transportation–related PA n (%)				0.155
<600 MET.min/week	109 (89.3)	25 (86.2)	95 (80.5)	
≥600 MET.min/week	13 (10.7)	4 (13.8)	23 (19.5)	
Housework–related PA n (%)				0.897
<600 MET.min/week	73 (59.8)	18 (62.1)	74 (62.7)	
≥600 MET.min/week	49 (40.2)	11 (37.9)	44 (37.3)	
Leisure–related PA n (%)				0.431
<600 MET.min/week	81 (66.4)	16 (55.2)	80 (67.8)	
≥600 MET.min/week	41 (33.6)	13 (44.8)	38 (32.2)	
Walking n (%)				0.812
<600 MET.min/week	85 (69.7)	19 (65.5)	78 (66.1)	
≥600 MET.min/week	37 (30.3)	10 (34.5)	40 (33.9)	
Moderate PA n (%)				0.006
<600 MET.min/week	80 (65.6)	14 (48.3)	91 (77.1)	
≥600 MET.min/week	42 (34.4)	15 (51.7)	27 (22.9)	
Vigorous PA n (%)				0.236
<600 MET.min/week	66 (54.1)	14 (48.3)	74 (62.7)	
≥600 MET.min/week	56 (45.9)	15 (51.7)	44 (37.3)	
Sitting time (hours/day)				0.018
Tertile 1 = ≤6.29 h	39 (35.1)	9 (37.5)	46 (42.2)	
Tertile 2 = 6.29-10 h	40 (36.0)	12 (50.0)	23 (21.1)	
Tertile 3 = >10 h	32 (28.8)	3 (12.5)	40 (36.7)	

$p<0.05$ was considered as significant; PA, Physical activity; MET, metabolic equivalent task; Total PA level (MET-minutes/week) = Total physical activity MET-minutes/week = sum of (Job PA + Transportation PA + Housework PA + Leisure PA MET-minutes/week scores).

Table S6 Association between PA levels & intensity (as measured by IPAQ) and BMI categories during lockdown

	BMI categories (Kg/m^2)				
	**<18.5 (n=62)**	**18.5-24.99 (n=462)**	**25-29.99 (n=208)**	**≥30 (n=61)**	***p*-value**
Total PA level n (%)					0.208
<600 MET.min/week	25 (40.3)	174 (37.7)	73 (35.1)	15 (24.6)	
≥600 MET.min/week	37 (59.7)	288 (62.3)	135 (64.9)	46 (75.4)	
Job–related PA n (%)					0.077
<600 MET.min/week	58 (93.5)	417 (90.3)	185 (88.9)	49 (80.3)	
≥600 MET.min/week	4 (6.5)	45 (9.7)	23 (11.1)	12 (19.7)	
Transportation–related PA n (%)					0.849
<600 MET.min/week	54 (87.1)	404 (87.4)	177 (85.1)	52 (85.2)	
≥600 MET.min/week	8 (12.9)	58 (12.6)	31 (14.9)	9 (14.8)	
Housework–related PA n (%)					0.290
<600 MET.min/week	39 (62.9)	315 (68.2)	127 (61.1)	38 (62.3)	
≥600 MET.min/week	23 (37.1)	147 (31.8)	81 (38.9)	23 (37.7)	
Leisure–related PA n (%)					0.793
<600 MET.min/week	42 (67.7)	312 (67.5)	134 (64.4)	43 (70.5)	
≥600 MET.min/week	20 (32.3)	150 (32.5)	74 (35.6)	18 (29.5)	
Walking n (%)					0.663
<600 MET.min/week	47 (75.8)	318 (68.8)	141 (67.8)	41 67.2)	
≥600 MET.min/week	15 (24.2)	144 (31.2)	67 (32.2)	20 (32.8)	
Moderate PA n (%)					0.544
<600 MET.min/week	43 (69.4)	335 (72.5)	146 (70.2)	39 (63.9)	

≥600 MET.min/week	19 (30.6)	127 (27.5)	62 (29.8)	22 (36.1)	
Vigorous PA n (%)					0.161
<600 MET.min/week	39 (62.9)	303 (65.6)	118 (56.7)	36 (59.0)	
≥600 MET.min/week	23 (37.1)	159 (34.4)	90 (43.3)	25 (41.0)	
Sitting time (hours/day)					0.400
Tertile 1 = ≤6.29 h	13 (22.8)	142 (33.3)	71 (37.4)	23 (42.6)	
Tertile 2 = 6.29-10 h	23 (40.4)	140 (32.8)	59 (31.1)	16 (29.6)	
Tertile 3 = >10 h	21 (36.8)	145 (34.0)	60 (31.6)	15 (27.8)	

p<0.05 was considered as significant; PA, Physical activity; MET, metabolic equivalent task; Total PA level (MET-minutes/week) = Total physical activity MET-minutes/week = sum of (Job PA + Transportation PA + Housework PA + Leisure PA MET-minutes/week scores).

Table S7 Association between PA levels & intensity (as measured by IPAQ) and type of PA during lockdown

	At-home workouts (n=193)	Outdoor activities (n=191)	At-home and outdoor activities (n=139)	No PA (n=272)	*p*-value
Total PA level n (%)					<0.001
<600 MET.min/week	60 (31.1)	37 (19.4)	22 (15.8)	169 (62.1)	
≥600 MET.min/week	133 (68.9)	154 (80.6)	117 (84.2)	103 (37.9)	
Job-related PA n (%)					0.013
<600 MET.min/week	174 (90.2)	159 (83.2)	125 (89.9)	252 (92.6)	
≥600 MET.min/week	19 (9.8)	32 (16.8)	14 (10.1)	20 (7.4)	
Transportation-related PA n (%)					<0.001
<600 MET.min/week	185 (95.9)	141 (73.8)	105 (75.5)	258 (94.9)	
≥600 MET.min/week	8 (4.1)	50 (26.2)	34 (24.5)	14 (5.1)	
Housework-related PA n (%)					<0.001
<600 MET.min/week	117 (60.6)	119 (62.3)	62 (44.6)	223 (82.0)	
≥600 MET.min/week	76 (39.4)	72 (37.7)	77 (55.4)	49 (18.0)	
Leisure-related PA n (%)					<0.001
<600 MET.min/week	127 (65.8)	112 (58.6)	59 (42.4)	235 (86.4)	
≥600 MET.min/week	66 (34.2)	79 (41.4)	80 (57.6)	37 (13.6)	
Walking n (%)					<0.001
<600 MET.min/week	156 (80.8)	89 (46.6)	73 (52.5)	230 (84.6)	
≥600 MET.min/week	37 (19.2)	102 (53.4)	66 (47.5)	42 (15.4)	
Moderate PA n (%)					<0.001

<600 MET.min/week	132 (68.4)	122 (63.9)	70 (50.4)	241 (88.6)	
≥600 MET.min/week	61 (31.6)	69 (36.1)	69 (49.6)	31 (11.4)	
Vigorous PA n (%)					<0.001
<600 MET.min/week	117 (60.6)	109 (57.1)	61 (43.9)	210 (77.2)	
≥600 MET.min/week	76 (39.4)	82 (42.9)	78 (56.1)	62 (22.8)	
Sitting time (hours/day)					<0.001
Tertile 1 = ≤6.29 h	50 (28.4)	81 (45.8)	48 (38.4)	66 (26.2)	
Tertile 2 = 6.29-10 h	74 (42.0)	54 (30.5)	48 (38.4)	85 (33.7)	
Tertile 3 = >10 h	52 (29.5)	42 (23.7)	29 (23.2)	101 (40.1)	

$p<0.05$ was considered as significant; PA, Physical activity; MET, metabolic equivalent task; Total PA level (MET-minutes/week) = Total physical activity MET-minutes/week = sum of (Job PA + Transportation PA + Housework PA + Leisure PA MET-minutes/week scores).

Figure S3 showing categories of Total and Leisure PA (≥ and <600 MET.min/week) according to type of PA during lockdown; participants who practiced both at-home and outdoor activities achieved the highest PA levels, $p<0.001$ for all

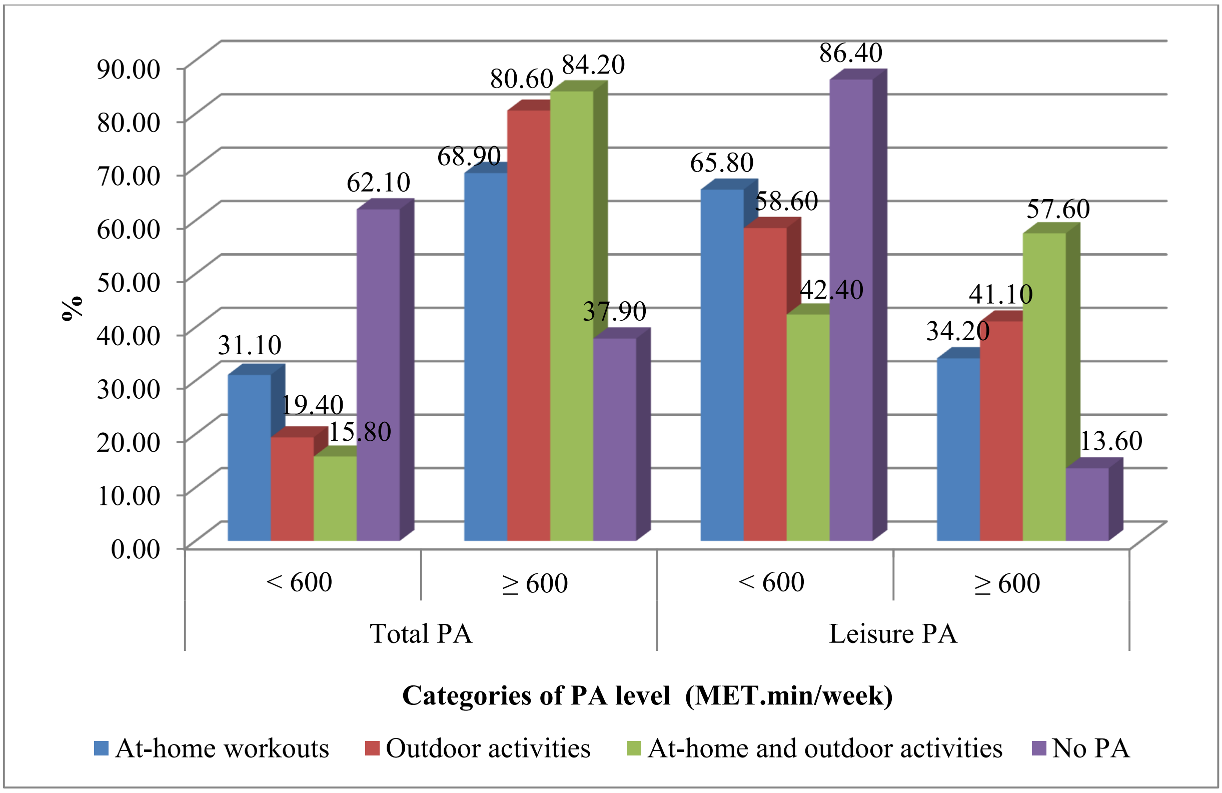

Table S8. Association between age and type of PA during lockdown

	**<23 years**	**23-40 years**	**≥40 years**	***p*-value**
**Type of PA during lockdown n(%)**				<0.001
At-home workouts	84 (27.8)	62 (25.7)	47 (18.7)	
Outdoor activities (walking, jogging, hiking)	51 (16.9)	47 (19.5)	93 (36.9)	
At-home and outdoor activities	62 (20.5)	48 (19.9)	29 (11.5)	
No PA	105 (34.8)	84 (34.9)	83 (32.9)	

$p<0.05$ was considered as significant; PA, Physical activity
